# Gastrointestinal function in intensive care patients: terminology, definitions and management. Recommendations of the ESICM Working Group on Abdominal Problems

**DOI:** 10.1007/s00134-011-2459-y

**Published:** 2012-02-07

**Authors:** Annika Reintam Blaser, Manu L. N. G. Malbrain, Joel Starkopf, Sonja Fruhwald, Stephan M. Jakob, Jan De Waele, Jan-Peter Braun, Martijn Poeze, Claudia Spies

**Affiliations:** 1 Clinic of Anaesthesiology and Intensive Care, University of Tartu, Puusepa 8, 51014 Tartu, Estonia; 2 Department of Intensive Care Medicine, University Hospital (Inselspital) and University of Bern, 3010 Bern, Switzerland; 3 Intensive Care Unit, Ziekenhuis Netwerk Antwerpen, ZNA Stuivenberg, Lange Beeldekensstraat 267, 2060 Antwerpen, Belgium; 4 Clinic of Anaesthesiology and Intensive Care, Tartu University Hospital, Puusepa 8, 51014 Tartu, Estonia; 5 Department of Anaesthesiology and Intensive Care Medicine, Medical University of Graz, Auenbruggerplatz 29, 8036 Graz, Austria; 6 Department of Critical Care Medicine, Ghent University Hospital and Ghent Medical School, De Pintelaan 185, 9000 Ghent, Belgium; 7 Department of Anaesthesiology and Intensive Care, Charité, Universitätsmedizin Berlin, Charitéplatz 1, 10117 Berlin, Germany; 8 Division of Traumatology, Department of Surgery, Maastricht University Medical Center, P. Debyelaan 25, 6202 AZ Maastricht, The Netherlands

**Keywords:** Gastrointestinal function, Failure, Symptoms, Feeding intolerance, Intensive care, Definitions, Classification

## Abstract

**Purpose:**

Acute gastrointestinal (GI) dysfunction and failure have been increasingly recognized in critically ill patients. The variety of definitions proposed in the past has led to confusion and difficulty in comparing one study to another. An international working group convened to standardize the definitions for acute GI failure and GI symptoms and to review the therapeutic options.

**Methods:**

The Working Group on Abdominal Problems (WGAP) of the European Society of Intensive Care Medicine (ESICM) developed the definitions for GI dysfunction in intensive care patients on the basis of the available evidence and current understanding of the pathophysiology.

**Results:**

Definitions for acute gastrointestinal injury (AGI) with its four grades of severity, as well as for feeding intolerance syndrome and GI symptoms (e.g. vomiting, diarrhoea, paralysis, high gastric residual volumes) are proposed. AGI is a malfunctioning of the GI tract in intensive care patients due to their acute illness. AGI grade I = increased risk of developing GI dysfunction or failure (a self-limiting condition); AGI grade II = GI dysfunction (a condition that requires interventions); AGI grade III = GI failure (GI function cannot be restored with interventions); AGI grade IV = dramatically manifesting GI failure (a condition that is immediately life-threatening). Current evidence and expert opinions regarding treatment of acute GI dysfunction are provided.

**Conclusions:**

State-of-the-art definitions for GI dysfunction with gradation as well as management recommendations are proposed on the basis of current medical evidence and expert opinion. The WGAP recommends using these definitions for clinical and research purposes.

**Electronic supplementary material:**

The online version of this article (doi:10.1007/s00134-011-2459-y) contains supplementary material, which is available to authorized users.

## Introduction

More than 10 years ago a round-table conference on gut dysfunction in critical illness concluded that intestinal function is an important determinant in the outcome of patients admitted to the intensive care unit (ICU), but that there is no objective and clinically relevant definition of gastrointestinal (GI) dysfunction in critical illness. In addition, it was stated that the definition developed in the future should grade the severity of dysfunction [1].

The problems in defining GI dysfunction start with defining GI function. Next to the digestive tract, the GI tract also carries out endocrine, immune and barrier functions. The clinical assessment of the impairment of these functions today is more intuitive than objective. Therefore, endocrine, immune and barrier dysfunctions will not be addressed in detail in the present paper.

Several studies have confirmed that GI symptoms are frequent in the ICU with up to 62% of patients exhibiting at least one GI symptom for at least 1 day [2–4]. There is also increasing evidence that development of GI problems is related to worse outcome in critically ill patients [2, 5–7].

Different definitions for separate GI symptoms have been used. The lack of markers for the measurement of GI function has suppressed studies in this field as well as the assessment of GI dysfunction as an organ failure. Although plasma citrulline and intestinal fatty acid binding protein have been proposed as possible markers for small bowel function [8], their clinical use in diagnosis and management of GI dysfunction is still unclear.

At least partly due to the lack of a formal definition and classification, treatment strategies for GI problems have been difficult to develop and are currently based on experience, rather than evidence.

There is increasing evidence that early protocolized and goal-oriented care can improve organ function and the patients’ outcome during critical illness [9–12]. Improving the definition of GI dysfunction as a part of the multiple organ dysfunction syndrome (MODS) and its derived sequential organ failure assessment score (SOFA) [13] will establish the base for setting up the bundle of preventive and therapeutic measures and support the development of novel treatment strategies.

For these reasons, the Working Group on Abdominal Problems (WGAP) as part of the Perioperative Intensive Care (POIC) section of the European Society of Intensive Care Medicine (ESICM) proposes a set of definitions and grading system of GI dysfunction in critical illness that are applicable both for clinical and research purposes.

## Methods

Several key elements were used as a starting point for defining acute GI organ failure. An organ failure was considered as a dichotomous event that is either present or absent, whereas organ dysfunction is a continuum of physiologic derangement [14]. The expression “GI dysfunction” is used to describe the large variety of GI symptoms (diarrhoea, vomiting) and diagnoses (gastroenteritis) outside of the ICU setting; therefore, the expression “acute GI injury” was introduced.

Current definitions and management recommendations (according to Table 1; [15]) were developed on the basis of the available evidence and current understanding of the pathophysiology. Definitions serve as expert opinion, with their reasoning given in each “rationale” subsection.

The working method is described in detail in the electronic supplementary file.

## Results

The WGAP suggests using the following terminology and definitions:

**Table 1:** Grading of the quality of evidence and strength of recommendations

Quality of evidence		Rationale
A	High	RCT or meta-analyses
B	Moderate	Downgraded RCTs or upgraded observational studies
C	Low	Well-done observational studies
D	Very low	Case series or expert opinion
Strength of recommendation		
Grade 1	Strong	We recommend
Grade 2	Weak	We suggest

*RCT* randomized controlled trial

**Fig. 1:**
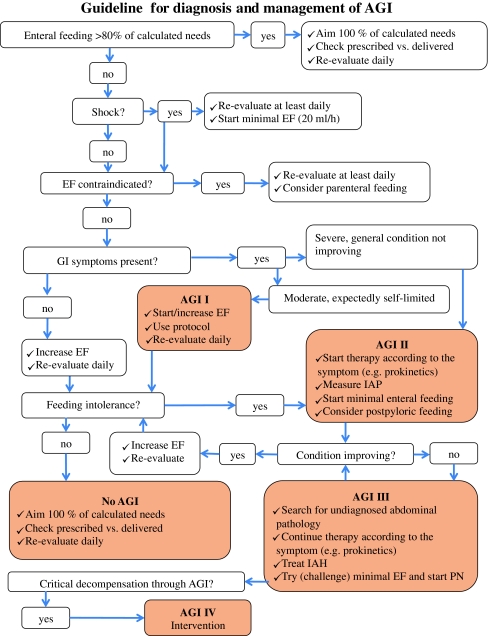
Schematic guideline for the management of patients with AGI.* EF* enteral feeding,* EN* enteral nutrition,* PN* parenteral nutrition

1. Gastrointestinal function
  The human GI tract has many functions including facilitating digestion to absorb nutrients and water, barrier control to modulate absorption of intraluminal microbes (and their products), endocrine and immune functions. Perfusion, secretion, motility and a coordinated gut–microbiome interaction are prerequisites for an adequate function [16].
  It needs to be underlined that because we currently lack the tool or marker to measure GI function we cannot reliably decide about normal GI function in the acute setting.
2. Acute gastrointestinal injury (AGI) and its different grades
  Acute GI injury (AGI) is malfunctioning of the GI tract in critically ill patients due to their acute illness.
  According to severity the following grades of AGI can be distinguished:
  2.1 AGI grade I (risk of developing GI dysfunction or failure)—the function of the GI tract is partially impaired, expressed as GI symptoms related to a known cause and perceived as transient.
*Rationale* Condition is clinically seen as occurrence of GI symptoms after an insult, which expectedly has temporary and self-limiting nature.
*Examples* Postoperative nausea and/or vomiting during the first days after abdominal surgery, postoperative absence of bowel sounds, diminished bowel motility in the early phase of shock.
*Management* The general condition is usually improving and specific interventions for GI symptoms are not needed, except the replacement of fluid requirements by intravenous infusions. Early enteral feeding, started within 24–48 h after the injury, is recommended [17, 18] (grade 1B). The use of drugs impairing GI motility (e.g. catecholamines, opioids) has to be limited whenever possible [19–22] (grade 1C).
  2.2 AGI grade II (gastrointestinal dysfunction)—the GI tract is not able to perform digestion and absorption adequately to satisfy the nutrient and fluid requirements of the body. There are no changes in general condition of the patient related to GI problems.
*Rationale* The condition is characterized by acute occurrence of GI symptoms requiring therapeutic interventions for achievement of nutrient and fluid requirements. This condition occurs without previous GI interventions or is more severe than might be expected in relation to the course of preceding abdominal procedures.
*Examples* Gastroparesis with high gastric residuals or reflux, paralysis of the lower GI tract, diarrhoea, intra-abdominal hypertension (IAH) grade I (intra-abdominal pressure (IAP) 12–15 mmHg), visible blood in gastric content or stool. Feeding intolerance is present if at least 20 kcal/kg BW/day via enteral route cannot be reached within 72 h of feeding attempt.
*Management* Measures to treat the condition and to prevent the progression to GI failure need to be undertaken (e.g. treatment of intra-abdominal hypertension [23], grade 1D; or measures to restore the motility function of GI tract, such as prokinetic therapy [24–26], grade 1C). Enteral feeding should be started or continued; in cases of high gastric residuals/reflux or feeding intolerance regular challenges with small amounts of enteral nutrition (EN) should be regularly considered (grade 2D). In patients with gastroparesis, initiation of postpyloric feeding should be considered in this state, when prokinetic therapy is not effective (grade 2D).
  2.3 AGI grade III (gastrointestinal failure)—loss of GI function, where restoration of GI function is not achieved despite interventions and the general condition is not improving.
*Rationale* Clinically seen as sustained intolerance to enteral feeding without improvement after treatment (e.g. erythromycin, postpyloric tube placement), leading to persistence or worsening of MODS.
*Examples* Despite treatment, feeding intolerance is persisting—high gastric residuals, persisting GI paralysis, occurrence or worsening of bowel dilatation, progression of IAH to grade II (IAP 15–20 mmHg), low abdominal perfusion pressure (APP) (below 60 mmHg). Feeding intolerance is present and possibly associated with persistence or worsening of MODS.
*Management* Measures to prevent worsening of GI failure are warranted (e.g. monitoring and targeted treatment of IAH [23], grade 1D). Presence of undiagnosed abdominal problem (cholecystitis, peritonitis, bowel ischaemia) should be excluded. The medications promoting GI paralysis have to be discontinued as far as possible [19–22] (grade 1C). Early parenteral feeding (within the first 7 days of ICU stay) supplementary to insufficient enteral nutrition is associated with higher incidence of hospital infections and should be avoided [27] (grade 2B). Challenges with small amounts of EN should be regularly considered (grade 2D).
  2.4 AGI grade IV (gastrointestinal failure with severe impact on distant organ function)—AGI has progressed to become directly and immediately life-threatening, with worsening of MODS and shock.
*Rationale* Situation when AGI has led to an acute critical deterioration of the general condition of the patient with distant organ dysfunction(s).
*Examples* Bowel ischaemia with necrosis, GI bleeding leading to haemorrhagic shock, Ogilvie’s syndrome, abdominal compartment syndrome (ACS) requiring decompression.
*Management* Condition requires laparotomy or other emergency interventions (e.g., colonoscopy for colonic decompression) for life-saving indications [28–30] (grade 1D). There is no proven conservative approach to resolve this situation.
    As differentiation of the acute GI problem from previously existing chronic condition might be very difficult, we suggest using the same definitions also in cases where the condition (e.g. GI bleeding, diarrhoea, etc.) might be due to a chronic GI disease (e.g. Crohn’s disease). In patients on chronic parenteral feeding, GI failure (equal to AGI III) should be considered chronically present, and no new acute interventions to restore function are indicated. However, monitoring of IAH and exclusion of the new acute abdominal problems should be performed similarly as in AGI grade III management.
  2.5 Primary and secondary AGI
    2.5.1 Primary AGI is associated with primary disease or direct injury to organs of the GI system (first hit).
*Rationale* Condition may usually be observed early (during the first day) after the insult to the GI system.
*Examples* Peritonitis, pancreatic or hepatic pathology, abdominal surgery, abdominal trauma, etc.
    2.5.2 Secondary AGI develops as the consequence of a host response in critical illness without primary pathology in the GI system (second hit).
*Rationale* Condition develops without direct insult to the GI tract.
*Examples* GI malfunction in a patient with pneumonia, cardiac pathology, non-abdominal surgery or trauma, postresuscitation state.
3. Feeding intolerance syndrome (FI)
  FI is a general term indicating intolerance of enteral feeding for whatever clinical reason (vomiting, high gastric residuals, diarrhoea, GI bleeding, presence of entero-cutaneous fistulas, etc.).
*Rationale* Diagnosis is based on complex clinical evaluation. There is no single clear-cut symptom or value that defines FI [31]. Several symptoms are commonly present.
  FI should be considered present if at least 20 kcal/kg BW/day via enteral route cannot be reached within 72 h of feeding attempt or if enteral feeding has to be stopped for whatever clinical reason. FI should not be considered as present if enteral feeding is electively not prescribed or is withheld/interrupted due to procedures.
  FI in special conditions: in a patient with postpyloric feeding, FI is defined similarly to gastric feeding. If a patient is not fed enterally due to the presence of entero-atmospheric fistulas, FI should be considered present. If the patient undergoes a surgical intervention for ACS or for changing of surgical dressings of an open abdomen, FI should be considered present immediately after surgery unless enteral feeding can be administered.
*Management* FI requires efforts to maintain/restore GI function: limiting the use of drugs impairing motility, application of prokinetics and/or laxatives [32–34] (grade 1C), and controlling IAP. Challenges with small amounts of EN should be regularly considered. In patients not tolerating enteral feeding, supplemental parenteral nutrition should be considered [35, 36] (grade 2D). Recent data suggest that delay for 1 week with parenteral nutrition enhances recovery when compared to early intravenous feeding [27] (grade 2B).
4. Intra-abdominal hypertension (IAH)
  4.1 IAH is present if IAP is found to be 12 mmHg or higher, confirmed by at least two measurements, 1–6 h apart [37].
*Rationale* Normal IAP is around 5–7 mmHg [38]. There are inherent variations and fluctuations in the IAP. IAH should also be considered present if the mean of the IAP measurements of the day is 12 mmHg or higher provided that at least four measurements were performed [39].
*Management* Monitoring of fluid resuscitation is necessary to avoid over-resuscitation [23] (grade 1C). Continuous thoracic epidural analgesia may decrease IAP in postoperative patients with primary IAH [40] (grade 2B). Nasogastric/colonic decompression is suggested for evacuation of intraluminal contents [23] (grade 2D). In patients with intraperitoneal fluids, percutaneous catheter decompression is recommended [23] (grade 1C). Elevation of head of bed above 20° should be considered as an additional risk for development of IAH [23] (grade 2C). Neuromuscular blockade decreases the IAP [41], but due to many undesirable effects it should be considered only in selected patients (grade 2C).
  4.2 Abdominal compartment syndrome (ACS) is defined as a sustained (minimally two standardized measurements, performed 1–6 h apart) increase in IAP above 20 mmHg with new onset organ failure [37].
*Management* Although decompression remains the only definite management for ACS, the exact indications and timing of this procedure still remain controversial [42]. Currently it is recommended (1) to perform surgical decompression as a life-saving intervention in patients with ACS that is refractory to other treatment options [28, 43] (grade 1D), and (2) to consider pre-emptive decompression at the time of laparotomy in patients who demonstrate multiple risk factors for IAH/ACS [23] (grade 1D). In most severe cases of ruptured abdominal aortic aneurysm or abdominal trauma the initial use of mesh closure avoids development of ACS [44, 45] (grade 1C).
5. Gastrointestinal symptoms
  5.1 Vomiting (emesis) is the occurrence of any visible regurgitation of gastric content irrespective of the amount.
*Rationale* Vomiting is commonly defined as the oral expulsion of GI contents resulting from contractions of gut and thoracoabdominal wall musculature [46]. Vomiting is contrasted with regurgitation, which is the effortless passage of gastric contents into the mouth [46]. In ICU patients the forcefulness of the act is often not detectable; therefore, regurgitation and vomiting should be assessed together.
*Management* Several guidelines for prevention and management of postoperative nausea and vomiting are available [47–51]. However, no studies have addressed management of vomiting in mechanically ventilated ICU patients; therefore, no specific recommendation can be given.
  5.2 Gastric residual volume could be considered high if a single volume exceeds 200 ml [52–54].
*Rationale* There is no sufficient scientific evidence or physiological ground to define precise values for high gastric residuals [53, 55]. Measurement of gastric residuals is neither standardized nor validated [56]. It has been suggested that gastric residual volume greater than 200 ml should prompt careful bedside evaluation, but automatic cessation of enteral nutrition solely on the basis on residual volumes of 200–500 ml should be avoided [53, 56, 57]. Despite the lack of scientific evidence, the members of the WGAP arbitrarily use total volumes of gastric residuals above 1,000 ml/24 h as a sign of abnormal gastric emptying, which requires specific attention.
*Management* Intravenous administration of metoclopramide and/or erythromycin is recommended for management of high gastric residuals, whereas cisapride is no longer approved [58] (grade 1B). Routine use of motility agents is not recommended [58] (grade 1A). Acupuncture stimulation may facilitate gastric empting in neurosurgical ICU patients [59] (grade 2B). Use of opioids and deep sedation should be avoided/reduced if possible. Cessation of gastric feeding is suggested if residual volumes exceed 500 ml per single measurement [57]. Here, postpyloric feeding should be considered [58] (grade 2D). Routine application of postpyloric feeding is not advocated [58] (grade 2D). Postpyloric feeding may cause severe small bowel dilatation and perforation in rare cases.
  5.3 Diarrhoea is defined as having three or more loose or liquid stools per day with a stool weight greater than 200–250 g/day (or greater then 250 ml/day) [60, 61].
*Rationale* Normal bowel frequency ranges from three times a week to three times a day. Secretory, osmotic, motor and exudative diarrhoea may be distinguished [61], but in the ICU it is often better to distinguish between disease-, food/feeding- and drug-related diarrhoea [61, 62].
*Management* Symptomatic therapy—replacement of fluids and electrolytes, haemodynamic stabilization and organ protection (e.g. correction of hypovolaemia to prevent impairment of renal function) forms the basic management [61, 63, 64] (grade 1D). At the same time, trigger mechanisms need to be discovered and when possible stopped (e.g. laxatives, sorbitol, lactulose, antibiotics) or treated (e.g. malabsorption, inflammatory bowel disease). Feeding-related diarrhoea in critically ill patients may require reduction of infusion rate, repositioning of feeding tube, or dilution of nutrition formula. Changing formula by adding soluble fibre prolongs transit time [61, 64–66] (grade1C). Only in cases of severe or recurrent *Clostridium difficile*-associated diarrhoea is oral vancomycin superior to metronidazole [67–69] (grade 2C).
  5.4 GI bleeding is any bleeding into the GI tract lumen, confirmed by macroscopic presence of blood in vomited fluids, gastric aspirate or stool.
*Rationale* Asymptomatic, endoscopically evident mucosal damage occurs in the majority of ICU patients [2]. Clinically evident GI bleeding reflecting considerable damage to GI mucosa may be seen in 5–25% of ICU patients [2]. Clinically important bleeding, defined as overt bleeding in association with haemodynamic compromise or the need for blood transfusions [70], occurs in 1.5–4% of mechanically ventilated patients [2, 70, 71].
*Management* In cases of clinically evident GI bleeding, the haemodynamic status dictates the approach. In cases of bleeding with haemodynamic instability endoscopy is the diagnostic tool of choice [72], but when bleeding is ongoing and massive, precluding adequate endoscopic assessment, angiography is appropriate (grade 2C). Early upper GI endoscopy (less than 24 h) is recommended [72, 73] (grade 1A), except for patients with acute variceal bleeding in whom a more expedite procedure (less than 12 h) should be considered [74] (grade 2C). Epinephrine injection can be used in combination with another method, such as clips, thermocoagulation or sclerosant injection [72] (grade 1A). Routine second endoscopy is not recommended, but in cases of rebleeding, a second attempt for endoscopic therapy is recommended [72] (grade 1A). In cases of a negative upper endoscopy with evidence of GI bleeding, colonoscopy should be performed, followed by small bowel exploration using push enteroscopy if colonoscopy is negative [75] (grade 2C). In cases of ongoing bleeding with negative endoscopies, abdominal surgery with intraoperative endoscopy or interventional radiology should be considered [76, 77] (grade 2C).
  5.5 Paralysis of lower GI tract (paralytic ileus) is the inability of the bowel to pass stool due to impaired peristalsis. Clinical signs include absence of stool for three or more consecutive days without mechanical obstruction. Bowel sounds may or may not be present.
*Rationale* Outside of the ICU, the terms constipation and obstipation include uncomfortable or infrequent bowel movements, hard stool and painful defecation. Because these symptoms may not be expressed in ICU patients, it is suggested to use the term paralysis of lower GI tract. A cut-off level of 3 days has been used in most of the epidemiological ICU studies [78, 79].
*Management* Inhibitory drugs for GI motility (e.g. catecholamines, sedatives, opioids) must be withdrawn if possible and conditions impairing motility (e.g. hyperglycemia, hypokalaemia) corrected [19–21] (grade 1C). Because of their delayed onset of action, laxative drugs must be started early or given prophylactically [24, 25] (grade 1D).
    Because of unknown long-term efficacy and safety the routine use of opioid antagonists cannot be recommended [80, 81] (grade 2B).
    Prokinetics like domperidone, metoclopramide and erythromycin are used to stimulate the upper GI tract (stomach and small bowel), whereas neostigmine stimulates small bowel and colonic motility [25, 30]. Despite the lack of well-controlled studies and sufficient evidence, we recommend a standardised approach in using prokinetics for management of motility disorders [24, 25] (grade 1D).
  5.6 Abnormal bowel sounds
*Rationale* Normal frequency of bowel sounds may range between 5 and 35 sounds/min [82]; the clinical significance of abnormal bowel sounds is not clear. No technique of auscultation has been proven to be superior [83]. The authors suggest auscultation for at least 1 min in two quadrants, repeated at least once within a tight time frame. Palpation of the abdomen before the auscultation may stimulate peristalsis causing subsequent bowel sounds that may not have been there otherwise [82].
    5.6.1 Absent peristalsis—no bowel sounds are heard at cautious auscultation.
*Rationale* Complete lack of bowel sounds is abnormal [83]. However, it should be recognized that presence of bowel sounds does not confirm normal motility, and that reoccurrence of bowel sounds does not correlate with improvement of paralysis.
    5.6.2 Hyperperistalsis is present if excessive bowel sounds are heard on auscultation.
*Rationale* Hyperperistalsis is a state of excessive motility of the digestive tract. It can be present during bowel obstruction occurring in parts of the bowel as attempts to overcome obstruction [84].
*Management* There are no special management suggestions for absent/abnormal bowel sounds.
  5.7 Bowel dilatation is present if colonic diameter exceeds 6 cm (greater than 9 cm for caecum) or small bowel diameter exceeds 3 cm, diagnosed either on plain abdominal X-ray or CT scan [85, 86].
*Rationale* Bowel dilatation is a common sign in obstruction at any level of the GI tract. Bowel dilatation may also appear without an obstruction; the terms toxic megacolon following colitis and acute colonic pseudo-obstruction or Ogilvie’s syndrome, are used to describe acute severe colonic dilatation.
*Management* Next to the correction of fluid and electrolyte imbalance, nasogastric decompression may be helpful [29, 87] (grade 1D), although routine usage of nasogastric tubes after elective laparotomy is not recommended [88] (grade 1A). After exclusion of mechanical obstruction, intravenous neostigmine could be considered in patients with a caecal diameter >10 cm and without improvement within 24 h [29, 89] (grade 2B). Colonoscopy is recommended for non-surgical decompression in patients with a caecal diameter >10 cm and no improvement after 24–48 h of conservative treatment [29, 87, 90] (grade 1C). Colonoscopic decompression is effective in up to 80%, but carries a certain morbidity/mortality risk [30]. Conservative treatment together with colonoscopy may be continued for 48–72 h unless the caecum is >12 cm wide [30, 91] (grade 2C). In cases of unresponsiveness to conservative treatment, surgery is indicated due to the threatening risk of perforation [29, 30] (grade 1D). Usage of a laparoscopic technique with thoracic epidural anaesthesia where appropriate enhances bowel function after abdominal surgery [92–94] (grade 1B), and may therefore prevent bowel dilatation.
6. Feeding protocols
  Decreased food intake and resulting malnutrition are independent risk factors for in-hospital mortality [95]. European Society for Parenteral and Enteral Nutrition (ESPEN) guidelines are available with recommendations for nutrition in intensive care [58]. Feeding protocols based on these guidelines should be implemented in every institution. Periods of interruption of enteral feeding due to various interventions in the hospital (surgery, diagnostic or therapeutic interventions, extubation) should be remembered and minimized [96, 97]. Daily assessment of adequacy of enteral nutrition is required.
7. A schematic guideline for the management of patients with AGI is presented in Fig. 1.

## Discussion

Terminology and definitions provided in the present paper were developed with the aim of providing clinical definitions which may be used in different ICUs and clinical situations.

Our working methods were similar to those commonly used for the consensus definitions and grading of evidence for the treatment recommendations. The main limitation of the current document is the lack of objective measures for GI function/dysfunction. As the evidence in this field is scarce, the definitions are largely based on expert opinion. Therefore, in case new established measures to assess GI function/dysfunction become available, proposed definitions need to be revised. The complete description of diagnostic procedures for conditions underlying AGI is not provided in the current manuscript, common clinical approach is presumed.

Our grading system is not based on a certain numeric variable and is not validated. With no doubt further research is needed to establish the measures of GI function that could be used in a reproducible manner for grading GI function. At present, the descriptions of the grades of AGI are complicated and the same grade of AGI may have different clinical expressions. It is likely that the score will in some extent be dependent on the treatment applied. In fact, other organ dysfunction scores (e.g. SOFA score) have also been developed first, and only validated afterwards. Moreover, the cardiovascular sub-score of SOFA [13], known to be the most performing among all the sub-scores, is defined as a mean arterial pressure and the usage/dosage of vasoactive/inotropic agents, where the last part is clearly dependent on the local treatment traditions.

Despite the many well-known limitations which have been restraining the development in this area for so long, we need to move forward, and we suggest to start with the definitions proposed in this paper.

## Summary

The terminology and definitions provided herein should allow better clinical communication as well as comparison between future studies. Defining the specific variables is the first step in a process towards better knowledge in this area. We propose a definition of acute gastrointestinal injury (AGI) with four grades of severity. AGI grade I stands for a self-limiting condition with increased risk of developing GI dysfunction or failure; AGI grade II (GI dysfunction) is a condition requiring interventions to restore GI function; AGI grade III (GI failure) is a condition when GI function cannot be restored with interventions; and AGI grade IV is dramatically manifesting GI failure, which is immediately life-threatening.

The WGAP of ESICM suggests using the proposed definitions until the results of a broader consensus are available. We encourage research to define explicit characteristics of GI function in critically ill patients.

## Electronic supplementary material

Below is the link to the electronic supplementary material.

Supplementary material 1 (DOC 25 kb)
