# Supplementary material for: Gastrointestinal function in intensive care patients: terminology, definitions and management. Recommendations of the ESICM Working Group on Abdominal Problems
Source: Intensive Care Med. 2012 Feb 7;38(3):384–94. doi: 10.1007/s00134-011-2459-y (PMC3286505; doi:10.1007/s00134-011-2459-y)
Supplement: Supplementary file 1 — Supplementary material 1 (DOC 25 kb) [file 134_2011_2459_MOESM1_ESM.doc]

*Working method*

The task to develop the definitions for GI dysfunction in ICU patients was formulated during the second WGAP meeting at the 22^nd^ Congress of the ESICM held in Vienna in October 2009. The writing committee was nominated during the same meeting. In early 2010, after reviewing the existing literature, the authors suggested a conceptual framework for standardizing the definitions. Thereafter, WGAP meetings were held at the 23^rd^ Congress of the ESICM in Barcelona, as well as at the 30^th^ and 31^st^ International Symposiums on Intensive Care and Emergency Medicine (ISICEM) held in Brussels in 2010 and 2011. Next to the conferences, the writing committee corresponded electronically, providing feedback to questions and issues raised during the conferences. Extensive literature review was performed and relevant evidence systematized and evaluated. Recommendations for management are based on the Grades of Recommendation, Assessment, Development and Evaluation (GRADE) system – a structured system for grading quality of evidence and strength of recommendation in clinical practice (Table 1). Consensus about the strength of each recommendation was reached through the discussion. Where available, evidence from existing guidelines of the European Society for Clinical Nutrition and Metabolism (ESPEN) and the World Society on Abdominal Compartment Syndrome (WSACS) was adopted and strength of recommendation was assessed by members of the writing committee.

This article serves as the final report of the ESICM WGAP consensus. The final version was approved by all members of the ESICM WGAP.
